# To sleep or not to sleep: An Italian Control-IQ-uestion

**DOI:** 10.3389/fendo.2022.996453

**Published:** 2022-12-12

**Authors:** Marta Bassi, Marina Francesca Strati, Valentina Andreottola, Maria Grazia Calevo, Giuseppe d’Annunzio, Mohamad Maghnie, Nicola Minuto

**Affiliations:** ^1^ Department of Neuroscience, Rehabilitation, Ophthalmology, Genetics, Maternal and Child Health, University of Genoa, Genoa, Italy; ^2^ Department of Pediatrics, IRCCS Istituto Giannina Gaslini, Genoa, Italy; ^3^ Epidemiology and Biostatistics Unit, IRCCS Istituto Giannina Gaslini, Genoa, Italy

**Keywords:** TIR (time in range), CGM (continuous glucose monitoring), Type 1 diabetes (T1D), tandem control-IQ, sleep, AHCL (Advanced Hybrid Closed Loop)

## Abstract

**Objective:**

Tandem Control-IQ is an advanced hybrid closed loop (AHCL) system with a Sleep Activity Mode to intensify glycemic control overnight. The aim of the study is to evaluate the effectiveness of using Sleep Mode or not among Tandem Control-IQ users.

**Research design and methods:**

We performed a retrospective Tandem Control-IQ data download for patients followed at IRCCS G. Gaslini Pediatric Diabetes Centre. We divided the patients into group 1 (Sleep Mode users) and group 2 (non-users) and compared their overall glycemic data, particularly during nighttime.

**Results:**

Group 1 (*n* = 49) does not show better nocturnal glycemic control as expected when compared with group 2 (*n* = 34). Group 2 shows a nighttime TIR% of 69.50 versus 66.25 (*p* = 0.20). Only the patients who do not use Sleep Mode and with sensor and automatic mode use ≥90% reached TIR >70% during nighttime, as well as lower nocturnal TAR% (18.80 versus 21.78, *p* = 0.05).

**Conclusions:**

This is the first study that evaluates the real-life effectiveness of the use of Sleep Mode in young patients with T1D. Control-IQ Sleep Activity Mode may not be as effective in Italian patients as in American patients due to the different habits.

## Introduction

The management of type 1 diabetes (T1D) has changed substantially in the last few years. New technologies allow the improvement of glycemic control by reducing the risk of hypoglycemia and hyperglycemia and decrease the rate of diabetes complications (1–3). Since the FDA approved the first hybrid closed loop (HCL) system, further advanced devices which integrate insulin infusion with continuous glucose monitoring (CGM) have been commercialized (4–6).

The goal of advanced hybrid closed loop (AHCL) technology is to reduce the burden of managing diabetes by automatically adjusting insulin delivery based on CGM data. Using CGM data, AHCL systems predict glucose values and adjust insulin delivery in order to keep glycemic values in a target range (7, 8).

The Tandem t:slim X2 insulin pump (Tandem Inc., San Diego, CA, USA) uses a Dexcom G6 sensor (Dexcom Inc., San Diego, CA, USA) and a closed loop algorithm (Control-IQ™) that automates basal insulin delivery and correction boluses, prevents and protects against hypoglycemia, and intensifies control overnight (9).

The Control-IQ system works based on well-defined target and treatment ranges with the aim of implementing the time spent in the recommended target range. When the predicted glucose value in the following 30 min is between 112.5 and 160 mg/dl, the pump delivers the basal insulin rate based on the active personal profile. When the predicted glucose value is <112.5 mg/dl, Control-IQ technology decreases personal insulin delivery rate and completely stops basal insulin delivery when predicted glucose values are below 70 mg/dl. When the predicted glucose value is above 160 mg/dl, the pump increases basal insulin delivery and delivers an automatic correction bolus if the predicted value is greater than 180 mg/dl. The system is able to deliver a maximum of one correction bolus per hour (reduced by 60% compared with the calculated).

Control-IQ technology has two integrated modes to optimize glycemic control during the night and during exercise; these modes can be activated and deactivated manually or scheduled by the patient. The Sleep Activity Mode works on a target range of 112.5–120 mg/dl instead of 112.5–160 mg/dl. When the predicted glucose value is >120 mg/dl, the pump increases the delivery of basal insulin, but it does not deliver correction boluses.

Currently, good glycemic control is defined on the basis of CGM data by the International Consensus as follows: time in range (TIR) (70–180 mg/dl) >70%, time below range (TBR) (<70 mg/dl) <4%, TBR (<54 mg/dl) <1%, time above range (TAR) (>180 mg/dl) <25%, and TAR (>250 mg/dl) <1% (10, 11).

Data from the first studies on the Control-IQ system in children and adults with type 1 diabetes have shown encouraging results in terms of glycemic outcomes and patient satisfaction (12, 13). Several multicenter randomized trials and real-life studies in children, adolescents, and adults demonstrated the efficacy of the Control-IQ technology when compared to the sensor-augmented pump, Basal-IQ technology, and other AHCL systems (14–20). The Control-IQ technology has been shown to be effective in terms of patient satisfaction, improvement of quality of life and quality of sleep for patients and parents, ease of use, and improvement of positive emotions (21–24).

Most studies have shown that the improvement in time in range is better overnight, which is consistent with the Control-IQ algorithm design (12–16, 19). Despite the evidence on the efficacy of the overnight system, there are no clinical studies evaluating the effectiveness of using or not using Sleep Mode among Tandem Control-IQ users.

The aim of this study was to compare glycemic control (globally and overnight) between Sleep Mode users and non-users in a cohort of children and young patients with type 1 diabetes using Control-IQ technology.

## Materials and methods

### Study design and study population

This was a retrospective study conducted in the Regional Reference Centre for Pediatric Diabetes, Istituto Giannina Gaslini, Genoa, Italy, a tertiary care pediatric hospital of Liguria, northwest Italy.

Patients were enrolled according to the following inclusion criteria: T1D diagnosis at least 1 year prior to the study, Tandem Control-IQ use for at least 1 month, and data download from February to May 2022 during an outpatient visit or a telemedicine visit. The exclusion criteria were as follows: percentage of use of automatic mode and/or sensor less than 80%, infections, or major changes in the usual lifestyle in the 14 days prior to data download (traveling, holidays, sickness).

### Data collection

During the first routine follow-up visit in which the inclusion criteria were met, the following data were collected for each patient: demographic data (sex, date of birth, age), age at clinical onset of T1D, duration of disease, time of use of the Control-IQ system, CGM data of the 14 days before the checkup, bolus time, and average consumption of carbohydrates (CHO) at dinner in the previous 14 days.

### Study outcomes

We divided the patients into two groups: group 1 (users of Sleep Mode for at least 6 h a night) and group B (non-users of Sleep Mode). During the Tandem Control-IQ training at our center, the Sleep Mode function is explained to all patients; then, they independently choose whether to set Sleep Mode or not. The following parameters were compared between the two groups: TIR, TAR, TAR >250 mg/dl, TBR, TBR <54 mg/dl, coefficient of variation (CV), standard deviation (SD), mean glucose value, glucose management indicator (GMI), percentage of sensor use, percentage of time in automatic mode, and percentage of time spent in Sleep Mode. We also compared the following data relating only to the night period (from 0 a.m. to 6 a.m.) between the two groups: TIR, TAR, TAR >250 mg/dl, TBR, TBR <54 mg/dl, CV, SD, and mean glucose value.

In addition, we decided to restrict the data analysis to patients who used sensor and automatic mode for a percentage of time greater than 90%, in order to select patients with the best possible use of the Control-IQ algorithm.

Considering the retrospective nature of the study, the informed consent form already signed by parents and/or patients at disease onset and renewed yearly, in which they agree on the use of clinical data for research purposes, was used. In addition, all parents and patients provided a specific informed consent form for the collection of data. The study was conducted in accordance with the Declaration of Helsinki and the International Conference on Harmonization Good Clinical Practice.

### Statistical analysis

Data are described as mean and SD or median and range for continuous variables and as absolute and relative frequencies for categorical variables.

Non-parametric analysis (Mann–Whitney *U* test) for continuous variables and the chi-square or Fisher’s exact test for categorical variables were used to measure differences between groups. *p*-values ≤0.05 were considered statistically significant, and all *p*-values were based on two-tailed tests. Statistical analysis was performed using SPSS for Windows (SPSS Inc., Chicago, IL, USA).

## Results

Data from a total of 110 T1D patients using Tandem Control-IQ (aged 4 to 35 years) were retrospectively collected at the IRCCS G. Gaslini Pediatric Diabetes Centre. We excluded 27 patients: 13 did not perform a visit or data download in the study period, 6 were diagnosed with diabetes in the previous year, 7 had become Tandem Control-IQ users less than a month prior to the beginning of the study period, and 1 used Sleep Mode for less than 6 h. We collected data of the remaining 83 T1D patients: 49 of these patients (group 1) used Sleep Mode and 34 (group 2) did not use it. Most patients of group 1 (*n* = 42) had scheduled Sleep Mode between 11 p.m. and 7 a.m.; the remaining 7 patients had scheduled it at different times between 10 p.m. and 8 a.m. and always for a duration of at least 6 h.

No significant differences were found in the clinical and demographic characteristics of the patients belonging to the two groups, with the exception of the percentage of nighttime sensor use, which was greater than 95% in both groups, as well as the duration of AHCL use (328.39 ± 111.34 days in group 1 and 181.50 ± 150.83 days in group 2, *p* = 0.001). Particularly, in our study population, the mean time of bolus for dinner was 8:17 p.m., and the mean number of carbohydrates consumed at dinner was 69.31 g; no significant differences were observed between the two groups for these meal parameters. The characteristics of the study population are summarized in **Table 1**.

**Table 1:** Comparison of the overall and nighttime (h 24–6) glycemic control of Sleep Activity Mode users (group 1) and non-users (group 2); analysis included patients using sensor and automatic mode ≥80% (*N* = 83).

	Sleep Activity users (*N* = 49)	Non-users (*N* = 34)	*p*-value
	*X* ± SD	*X* ± SD	
Gender, M (%)	23 (46.9)	19 (55.9)	0.50
Age (years)	17.09 ± 6.01	18.51 ± 8.27	0.42
Duration of disease (years)	8.29 ± 5.76	12.13 ± 8.41	0.06
Sensor use (%)	94.61 ± 3.24	94.03 ± 2.91	0.17
Nighttime (h 24–6) sensor use (%)	97.75 ± 3.29	96.19 ± 4.10	**0.05**
Time in automatic mode (%)	94.84 ± 3.89	94.44 ± 3.90	0.47
Time in Sleep Activity Mode (%)	32.71 ± 5.67	–	
Dinner CHO consumption (g)	67.02 ± 23.93	72.62 ± 23.55	0.27
Bolus time for dinner (hh:mm—p.m.)	8:19 ± 37:56	8:13 ± 36:36	0.24
Duration of AHCL use (days)	328.39 ± 111.34	181.50 ± 150.83	**0.001**
TIR (%)	67.80 ± 12.13	70.79 ± 11.07	0.20
TAR (%)	21.59 ± 6.78	19.26 ± 6.19	0.08
TAR >250 mg/dl (%)	8.61 ± 7.63	7.59 ± 6.23	0.70
TBR (%)	1.63 ± 1.72	1.88 ± 1.72	0.26
TBR <54 mg/dl (%)	0.50 ± 0.77	0.56 ± 0.77	0.73
Mean glucose (mg/dl)	159.02 ± 21.09	155.24 ± 19.24	0.30
GMI (%)	7.12 ± 0.62	7.06 ± 0.51	0.59
SD (mg/dl)	56.04 ± 12.31	55.21 ± 10.78	0.89
CV (%)	35.10 ± 5.22	35.41 ± 4.07	0.35
Nighttime TIR (%)	66.25 ± 15.45	69.50 ± 13.55	0.51
Nighttime TAR (%)	23.61 ± 11.01	21.96 ± 8.30	0.88
Nighttime TAR >250 mg/dl (%)	8.35 ± 8.16	7.28 ± 7.10	0.62
Nighttime TBR (%)	1.14 ± 2.01	1.06 ± 1.54	0.96
Nighttime TBR <54 mg/dl (%)	0.56 ± 1.28	0.47 ± 0.83	0.87
Nighttime mean glucose (mg/dl)	161.41 ± 23.61	160.62 ± 21.69	0.91
Nighttime SD (mg/dl)	52.77 ± 13.07	49.41 ± 11.72	0.23
Nighttime CV (%)	32.82 ± 6.60	30.57 ± 4.89	0.09

CV, coefficient of variation; GMI, glucose management indicator; SD, standard deviation; TIR, time in range (70–180 mg/dl); TAR, time above range (>180 mg/dl); TAR >250 mg/dl, time above range (>250 mg/dl); TBR, time below range (<70 mg/dl); TBR <54 mg/dl, time below range (<54 mg/dl) bold = statistically significant.

The differences in overall and nocturnal glycemic control between the two groups are shown in **Table 1**. Group 1 had a similar TIR% compared with group 2 both overall (67.80 ± 12.13 versus 70.79 ± 11.07, *p* = 0.20) and during nighttime (66.25 ± 15.45 versus 69.50 ± 13.55, *p* = 0.51).

Limiting the analysis to patients with percentage of time of sensor use and automatic mode use ≥90% (*N* of patients = 71), data confirmed a similar TIR% in group 1 compared with group 2 overall (68.00 ± 12.81 versus 71.97 ± 9.58, *p* = 0.20) and during nighttime (66.52 ± 15.76 versus 70.77 ± 12.46, *p* = 0.43). A statistically significant difference between the two groups in terms of TAR% (21.78 ± 7.10 in group 1 versus 18.80 ± 5.94 in group 2, *p* = 0.05) was observed (**Table 2**).

**Table 2:** Comparison of the overall and nighttime (h 24-6–) glycemic control of Sleep Activity Mode users (group 1) and non-users (group 2); analysis included patients using sensor and automatic mode ≥90% (*N* = 71).

	Sleep Activity users (*N* = 41)	Non-users (*N* = 30)	*p*-value
	*X* ± SD	*X* ± SD	
Gender, M (%)	17 (41.5)	17 (56.7)	0.24
Age (years)	16.76 ± 6.01	18.60 ± 8.35	0.31
Duration of disease (years)	8.34 ± 6.01	11.71 ± 8.50	0.16
Sensor use (%)	95.49 ± 2.13	94.67 ± 2.06	0.06
Nighttime sensor use (%)	98.39 ± 2.46	97.13 ± 2.58	**0.05**
Time in automatic mode (%)	96.22 ± 2.04	95.47 ± 2.47	0.20
Time in Sleep Activity Mode (%)	32.56 ± 6.05	–	
TIR (%)	68.00 ± 12.81	71.97 ± 9.58	0.20
TAR (%)	21.78 ± 7.10	18.80 ± 5.94	**0.05**
TAR >250 mg/dl (%)	8.59 ± 8.08	6.84 ± 4.82	0.71
TBR (%)	1.39 ± 1.38	1.93 ± 1.78	0.10
TBR <54 mg/dl (%)	0.35 ± 0.53	0.57 ± 0.80	0.92
Mean glucose (mg/dl)	159.78 ± 21.86	153.03 ± 15.91	0.15
GMI (%)	7.14 ± 0.63	7.00 ± 0.47	0.30
SD (mg/dl)	55.07 ± 12.61	54.10 ± 9.35	0.89
CV (%)	34.27 ± 4.80	35.23 ± 4.00	0.13
Nighttime TIR (%)	66.52 ± 15.76	70.77 ± 12.46	0.43
Nighttime TAR (%)	24.10 ± 11.39	21.25 ± 8.26	0.56
Nighttime TAR >250 mg/dl (%)	7.97 ± 8.14	6.35 ± 6.08	0.56
Nighttime TBR (%)	1.00 ± 2.03	1.13 ± 1.62	0.60
Nighttime TBR <54 mg/dl (%0)	0.35 ± 0.90	0.50 ± 0.86	0.43
Nighttime mean glucose (mg/dl)	162.02 ± 23.60	158.63 ± 19.99	0.62
Nighttime SD (mg/dl)	51.51 ± 13.15	48.26 ± 10.65	0.30
Nighttime CV (%)	31.86 ± 6.49	30.28 ± 4.80	0.27

CV, coefficient of variation; GMI, glucose management indicator; SD, standard deviation; TIR, time in range (70–180 mg/dl); TAR, time above range (>180 mg/dl); TAR >250 mg/dl, time above range (>250 mg/dl); TBR, time below range (<70 mg/dl); TBR <54 mg/dl, time below range (<54 mg/dl) bold = statistically significant.

No statistically significant differences in terms of TBR, CV, SD, mean glucose, and GMI were found between the two groups in either the original or the restricted analysis (**Tables 1**, **2**). Further stratifying the analysis between age groups (<18 and ≥18 years), no significant differences were found for all the nocturnal parameters analyzed.

**Figure 1:**
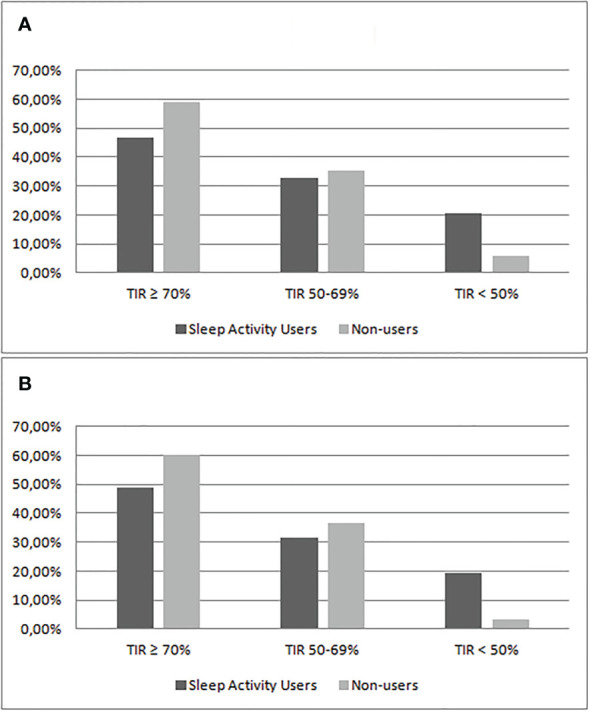
Comparison of the percentage of patients divided by categories of nighttime TIR between Sleep Activity Mode users (group 1) and non-users (group 2). The analysis included patients using sensor and automatic mode ≥80% **(A)** or ≥90% **(B)**.

Comparing the patients’ nighttime TIR (TIR ≥70%, TIR 50%–70%, and TIR <50%), data showed that the percentage of patients that reach the recommended target of TIR ≥70% is 46.9% in group 1 and 58.8% in group 2. Patients who have a nighttime TIR lower than 50% are 20.4% of group 1 and 5.9% of group 2. Restricting the analysis to patients who used automatic mode and sensor for more than 90% of the time, 19.5% of the patients in group 1 and 3.3% in group 2 (*p* = 0.09) had nocturnal TIR <50% (**Tables 3**, **4**; **Figure 1**).

**Table 3:** Comparison by category of nighttime TIR between Sleep Activity Mode users (group 1) and non-users (group 2); analysis included patients using sensor and automatic mode ≥80% (*N* = 83).

	Sleep Activity users (*N* = 49)	Non-users (*N* = 34)	*p*-value
	*N* (%)	*N* (%)	
TIR ≥70%	23 (46.9)	20 (58.8)	0.14
TIR 50%–69%	16 (32.7)	12 (35.3)	
TIR <50%	10 (20.4)	2 (5.9)	

**Table 4:** Comparison by category of nighttime TIR between Sleep Activity Mode users (group 1) and non-users (group 2); analysis included patients using sensor and automatic mode ≥90% (*N* = 71).

	Sleep Activity users (*N* = 41)	Non-users (*N* = 30)	*p*-value
	*N* (%)	*N* (%)	
TIR ≥70%	20 (48.8)	18 (60.0)	0.09
TIR 50%–69%	13 (31.7)	11 (36.7)	
TIR <50%	8 (19.5)	1 (3.3)	

## Discussion

The aim of this study was to compare real-life glycemic control data between Tandem Control-IQ Sleep Mode Users and non-users. To the best of our knowledge, this is the first study to compare the overnight effectiveness of the Tandem Control-IQ Sleep Mode compared with the Standard Control-IQ algorithm.

The Control-IQ system has been shown to be effective in glycemic control and has been appreciated by patients since the first studies on children and adults with type 1 diabetes (12, 13). Several multicenter randomized trials in children, adolescents, and adults demonstrated the efficacy of Control-IQ compared with sensor-augmented pumps, showing an improvement in TIR without increasing hypoglycemia (14–16). A recent study in children demonstrates an improvement of TIR with Control-IQ in comparison with Basal-IQ, a predictive low-glucose suspend (PLGS) algorithm (17). A single multicenter study that compared AHCL systems currently approved for the pediatric population showed the non-inferiority of the efficacy of Tandem Control-IQ in reaching glycemic targets compared with the other systems (18). Recent studies on the real-world use of the Tandem Control-IQ system confirmed the conclusions reached by the pivotal trials and previous studies. The use of Control-IQ technology increased time in range at 12 months in a sample of 7,813 patients (19) and at 6 months in a sample of 191 youth patients with type 1 diabetes (20).

Most studies on the effectiveness of the Control-IQ System included additional analysis focusing on the overnight period; all of these studies have shown that the algorithm is more effective on TIR during nighttime (12–16, 19). Forlenza et al. observed significative improvement of TIR overnight (from 11 p.m. to 7 a.m.) in the Control-IQ group compared with the sensor-augmented pump (SAP) group (Control-IQ: 74.9%–10.1% *vs*. SAP: 49.6%–18.8%; *p* = 0.001), with an overall TIR of 71.0% in Control-IQ users (12). In the first randomized multicenter trial of closed loop control (CLC) in T1D, Brown et al. showed that TIR was 70% in the closed loop group and 59% in the control group during the daytime (6 a.m. to midnight) and was 76% and 59%, respectively, during the nighttime (midnight to 6 a.m.) (13). Comparing CLC with SAP, Breton et al. observed a daytime (6 a.m. to midnight) TIR of 63% in the closed loop group and 56% in the control group, and the corresponding values during the nighttime (midnight to 6:00 a.m.) were 80% and 54% (14). Kanapka et al. also observed a better improvement in TIR overnight (midnight to 6 a.m.), while Isganaitis et al. showed an improvement in TIR in the Control-IQ group compared with the SAP group especially between 1 a.m. and 8 a.m. (+19% of TIR at night and +11% during the day, *p* < 0.0001) (15, 16). Recently, Breton et al. showed a profound TIR increase at night, reaching a median >90% between 4 and 7 a.m. in T1D Control-IQ users in a 1-year real-world study (19).

Despite the evidence on the efficacy of the system overnight, to date, there are no studies that evaluate the effectiveness of using Sleep Mode or not among Tandem Control-IQ users.

We decided to download the data during the visits that took place between February and May 2022. The choice to download data only in a specific time window derives from the need to avoid as much as possible substantial differences in the life habits of patients related to the pandemic situation and to the seasonal habits and to exclude Italian prolonged periods of holidays (e.g., Christmas or summer holidays); in particular, schools, sports, and extracurricular activities were open in Italy during the study period. Patients followed at the IRCCS G. Gaslini Pediatric Diabetes Centre are both children and young adults (up to 35 years), and this is the reason for the age range of the study population. Despite the wide spectrum of the age of the patients included, stratifying the analysis by age, no significant differences emerged in the parameters analyzed compared with the entire population.

We defined nighttime as the period between midnight and 6 a.m., according to most of the studies that evaluated the effectiveness of Tandem Control-IQ overnight (13–15); all the scheduled Sleep Mode set by the group 1 patients included this time range. The study had predefined inclusion criteria of time in connectivity in closed loop control and CGM of at least 80% overall and during nighttime; a lower percentage of use of the closed loop technology would not allow to evaluate the algorithm and to compare the night mode with the standard mode adequately (8).

Data showed that the use of Sleep Mode does not significantly improve nighttime glycemic control and that the group of non-users surprisingly has a similar overnight TIR (69.50 ± 13.55 versus 66.25 ± 15.45). Further restricting the analysis to patients with an automatic insulin delivery time and sensor use greater than 90%, we also observed that only the group of non-users of Sleep Mode (TIR% 70.77 ± 12.46) reached the recommended TIR (11), and TAR% is surprisingly and significantly reduced in this group (18.80 ± 5.94 versus 21.78 ± 7.10, *p* = 0.05). The overnight TBR% did not significantly increase in group 2 (TBR 1.06 ± 1.54 versus 1.14 ± 2.01). The mean duration of use of AHCL was longer in patients belonging to group 1 (about 6 versus 11 months in group 2); therefore, the experience of using AHCL can be considered a factor in favor of group 1. These data demonstrate the non-inferiority and safety of non-use of the Control-IQ Sleep Mode (**Tables 1**, **2**).

Furthermore, by stratifying the population by TIR groups, we observed that in patients who do not use the Sleep Mode, the percentage of patients who reached the recommended target of TIR ≥70% is higher compared with users (58.8% versus 46.9%), and the percentage of patients with a nighttime TIR lower than 50% is lower compared with those who use the Sleep Mode (5.9% versus 20.4%). Restricting the analysis to patients using automatic mode and sensor for more than 90% of the time, patients with nocturnal TIR <50% were less in the non-user group (3.3% versus 19.5%) (**Tables 3**, **4** and **Figure 1**). These data, despite not reaching statistical significance, once again underline the non-efficacy of the Sleep Mode in our sample of young Italian patients.

The Sleep Mode has a narrower target range (112.5–120 mg/dl) to ensure optimal glucose values during the night and has been shown to perform brilliantly in system efficacy studies and in comparison with SAP. Nevertheless, if Sleep Mode is activated, no corrective boluses are delivered; on the one hand, this feature guarantees the safety of the algorithm during the night; on the other hand, the increase in the basal rate alone may sometimes not be sufficient to quickly bring glucose values back to target values. The Sleep Mode may be more effective when bedtime glucose value is in the target range, while in the case of post-dinner hyperglycemia or the consumption of foods with high-fat content, it may have more difficulty in bringing glucose values back to the target. This particular feature assumes great relevance in a country like Italy, where dinner is served late (usually from 8 p.m. to 9 p.m.), often rich in carbohydrates, and children often go to sleep shortly after dinner consumption. These Italian habits are very different from the American ones, on the basis of which the Control-IQ standard algorithm and activity modes were probably created. All of the system efficacy studies on nocturnal glycemic control were also performed in the USA (12–16, 19).

This is the first study to evaluate the real-life effectiveness of the use of Sleep Mode compared with the Control-IQ standard mode in young patients with T1D, and the results are certainly interesting and challenging. The Control-IQ Sleep Mode may not be effective in Italian patients due to the different habits compared with American patients. The limitations of the study included the low number of patients, the retrospective model of the study, and the real-life nature of the study, which allow us to evaluate the effectiveness of the system in the daily life of patients but can give less uniformity in lifestyle habits. Further studies with a greater number of patients in uniform settings such as school camps or group activities that analyze the effectiveness of Sleep Mode in relation to the consumption of certain foods are certainly needed.

## Data availability statement

The original contributions presented in the study are included in the article/supplementary material. Further inquiries can be directed to the corresponding authors.

## Ethics statement

Ethical review and approval was not required for the study on human participants in accordance with the local legislation and institutional requirements. Written informed consent to participate in this study was provided by the participants’ legal guardian/next of kin.

## Author contributions

MB designed the study and wrote the manuscript. MS researched the data and wrote the manuscript. VA researched the data. MC did the statistical analysis. Gd’A reviewed the manuscript and contributed to the discussion. MM reviewed the manuscript and contributed to the discussion. NM designed the study and contributed to the discussion. All authors contributed to the article and approved the submitted version.
